# Electrospun Tubing as Shaping Material for Central Vessels in Perfusable, Vascularized Constructs

**DOI:** 10.1002/mco2.70985

**Published:** 2026-09-08

**Authors:** Daria Wehlage, Mia Göcht, Andrea Ehrmann, Birgit Andrée

**Affiliations:** ^1^ Leibniz Research Laboratories for Biotechnology and Artificial Organs (LEBAO) Department of Cardiothoracic Transplantation and Vascular Surgery Hannover Medical School Hannover Germany; ^2^ Faculty of Engineering and Mathematics Bielefeld University of Applied Sciences and Arts Bielefeld Germany

1

Dear Editor:

Tissue engineering (TE) is a promising technique for the reconstruction of failing tissues and organs. The major obstacle in TE is the nourishment of cells in dense tissues of clinically relevant dimensions requesting the implementation of a perfusable vascular system lined with endothelial cells (ECs). Although substantial progress has been achieved in the last decades, the generation of a functional vascular tree with small arteries for surgical anastomosis and capillaries for nourishment of cells is still a major hurdle. Recently, a few attempts for expanding vascularization from a central tube into a surrounding hydrogel were reported [1, 2]. However, their structural and dimensional limitations preclude direct clinical application.

Here, we envision electrospinning for the fabrication of tubes with the dimensions of small arteries by employing poly‐ε‐caprolacton (PCL), which was identified as an excellent material for the fabrication of small vascular grafts. In our study, we evaluate highly porous, electrospun PCL (ePCL) tubes as a supporting and shaping material for hydrogels containing ECs and human adipose‐tissue‐derived stromal cells (hASCs) (Figure 1A), which enable the self‐assembly of stable, capillary‐like EC networks with hollow structures demonstrated previously by us [3].

**FIGURE 1:**
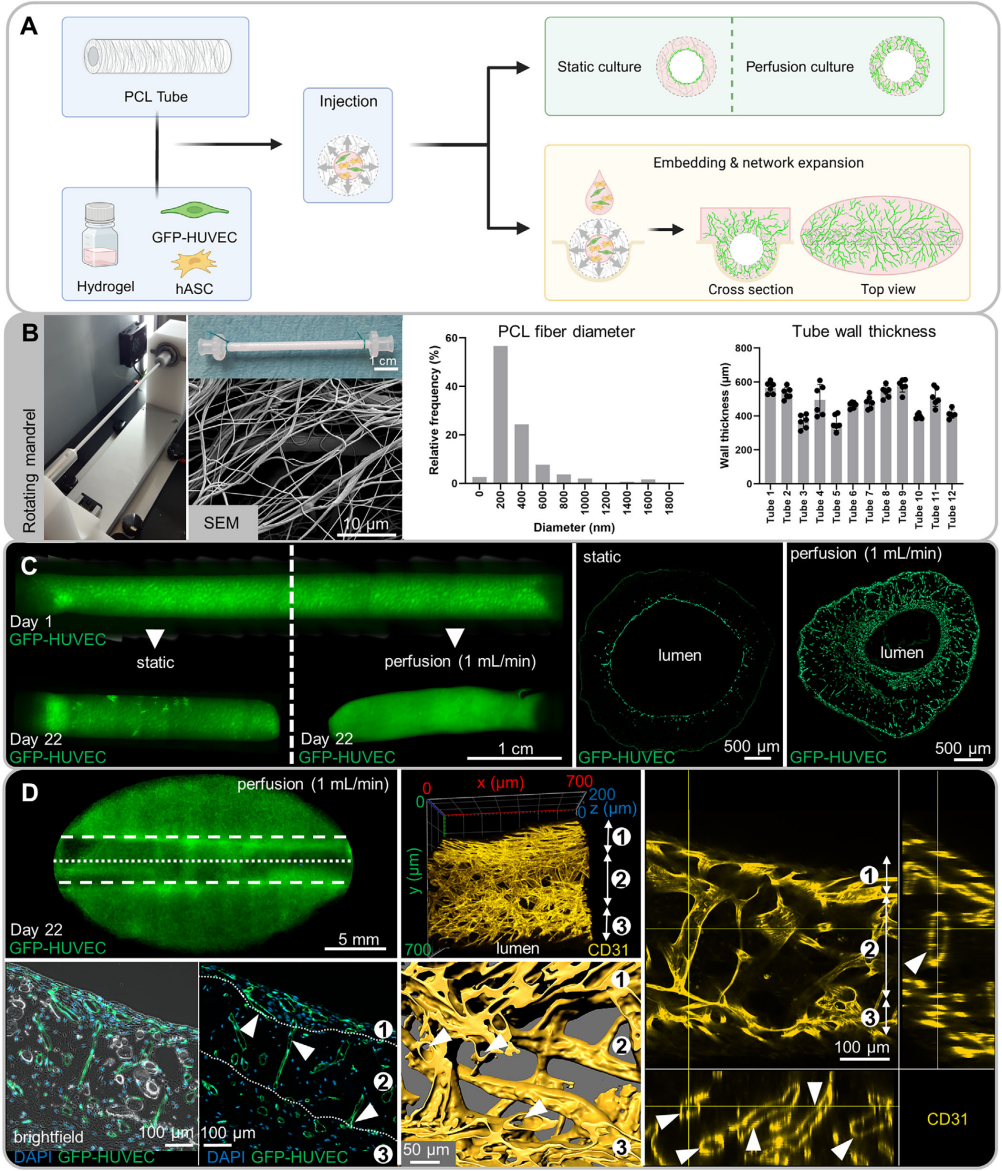
Successful generation of large vascular structures by combining an electrospun PCL (ePCL) tube and hydrogel‐based self‐assembly of endothelial cells through cultivation under perfusion. (A) Schematic outline. A hydrogel based on rCOL containing GFP‐HUVECs and hASCs is injected into an ePCL tube, which was either cultivated under static or perfused conditions. For the generation of large networks, an ePCL tube was placed in the groove of a metal plate laid out with SIS. After injection of the cell containing hydrogel into the ePCL tube, an oval‐shaped silicon mold was positioned on the tube to define the casting area for an additional cell containing hydrogel for network expansion. (B) Fabrication and characterization of ePCL tubes. ePCL tubes were produced using a rotating mandrel and were equipped with Luer hose connectors (left). SEM images were taken and the distribution of the PCL fiber diameter was quantified (middle). Wall thickness was assessed from cross‐sectional images of 12 individual tubes, with three measurements performed per cross‐section (right). Scale bars: 1 cm; 2 µm. (C) Cultivation of a hydrogel‐injected ePCL tube under perfusion resulted in the formation of an EC network throughout the PCL matrix in comparison to limited infiltration of ECs under static conditions. Top view of an ePCL tube injected with a hydrogel containing GFP‐HUVECs (green) and hASC after 24 h of static pre‐cultivation and before bisection (top left), followed by top views of each half of the bisected PCL tube after 22 days of culture under static or perfused conditions (bottom left). Cross‐sections of the respective PCL tubes revealing the distribution of GFP‐HUVECs under static conditions (middle) and perfusion (right). Scale bars: 1 cm; 500 µm. (D) Integration of a hydrogel‐injected PCL tube into a larger hydrogel construct leads to a connected vascularized structure. Top view of a construct combining a hydrogel‐injected PCL tube with a hydrogel containing GFP‐HUVECs (green) and hASCs cast on top at Day 22 of cultivation under perfusion (top left). The dashed lines indicate the position of the PCL tube, while the dotted line indicates the plane for the longitudinal section displayed on the bottom left, showing a merged image of DAPI (blue), GFP‐HUVECs (green), and brightfield (left); without brightfield (right). White arrowheads indicate GFP‐HUVEC networks extending from the PCL tube into the surrounding hydrogel. Dashed lines indicate the location of the PCL‐tube wall. Whole mount immunofluorescence staining for CD31 (yellow) and a 3D reconstruction of retrieved confocal images demonstrate the extensive network in a 260 µm slice (top middle). A higher magnification image of a 3D surface reconstruction of a 100 µm thick slice from the above‐shown confocal stack demonstrates the presence of a hollow structure extending through the whole depth of the slice (white arrowheads) and a connected vascularized structure through all layers (middle bottom). Orthogonal views of a confocal image confirm the presence of hollow structures (right). White arrowheads indicate CD31 positive structures with lumen. (1): covering hydrogel, (2): PCL‐tube wall, (3): luminal cell accumulation. Scale bars: 5 mm; 100 µm.

PCL was electrospun onto a rotating mandrel generating mechanically stable tubes with a length of 60 mm and an inner diameter of 3 mm (Figure 1B, left). ePCL tubes exhibited a mean fiber diameter of 346 nm (Figure 1B, middle), determined via SEM images, and an average wall thickness of (476 ± 83) µm (Figure 1B, right) with a porosity of (79.5 ± 7.3)%. Initially, a hydrogel based on rat tail collagen (rCOL) containing GFP‐labeled human umbilical vein endothelial cells (GFP‐HUVECs) and hASCs was cast onto such ePCL tubes. But neither under static conditions nor under perfusion a fully vascularized tube was accomplished. Therefore, injection of the hydrogel into the lumen by applying gentle pressure to force the hydrogel into the porous structure of the PCL tube was evaluated (Figure 1A). To maintain the lumen of the tube, a metal rod was inserted, which was removed after polymerization of the hydrogel. After 24 h, the whole length of the tube was homogeneously populated with GFP‐HUVECs (Figure 1C, top left). The tube was bisected and one half was subjected to perfusion, while the other half was maintained under static conditions. After 22 days of static cultivation, no macroscopically visible EC network formation was observed on the exterior of the ePCL tube (Figure 1C, bottom left), while under perfusion, we observed a dense interwoven EC network on the exterior of the PCL tube (Figure 1C, bottom left). This observation was confirmed in cross‐sections revealing the distribution of GFP‐HUVECs limited to the luminal surface with only a few cells detected within the wall of the ePCL tube under static conditions (Figure 1C, middle). In contrast, cultivation under perfusion resulted in an extensive EC network penetrating the whole tube wall extending from the lumen to the outer perimeter of the tube (Figure 1C, right), probably due to ECs being exposed to shear stress. Although the calculated shear stress is rather small in our setting (0.06 dyn/cm^2^), it was recently demonstrated that the magnitude of shear stress is crucial with low shear stresses being beneficial for vascular network formation [4]. Interestingly, only during cultivation under perfusion, substantial collagen deposition (data not shown) and narrowing of the lumen was observed (Figure 1C, right) suggesting that the low shear stress in our system also enhanced cellular proliferation, migration, and matrix deposition. To prevent this excessive proliferation, growth stimuli could be reduced by cultivation in serum‐free medium developed by us that permits the formation of EC networks [5]. Taken together, the injection of a cell‐containing hydrogel into a PCL support matrix, followed by cultivation under perfusion, resulted in an EC network extending throughout the entire wall of the ePCL tube, successfully generating a pre‐vascularized main tubing.

We subsequently evaluated such tubes for their integration into larger vascularized constructs. Therefore, a metal mold with a groove was laid out with decellularized porcine small intestinal submucosa as supporting matrix. A pre‐vascularized tube was placed into the groove and covered with an additional cell‐containing rCOL hydrogel. During this procedure, it became apparent that interfacial barrier formation and subsequent detachment of the layers was hampering the formation of large vascularized structures. In fact, gel–gel interfaces are known to form quickly during sequential casting of hydrogels, which can limit cell migration across the interface depending on the cell type. However, when a pure ePCL tube was placed and secured in the groove of the metal mold, followed by the injection of a cell‐containing hydrogel and directly casting a second hydrogel on top of the tube (Figure 1A, yellow box), no interface formation was observed. In a top view, an extensive network of GFP‐HUVECs was visible after 22 days of cultivation under perfusion (Figure 1D, top left). In sections, the distribution of the GFP‐HUVECs throughout the whole construct forming vessel‐like hollow structures was demonstrated (Figure 1D, bottom left). Most importantly, the formation of vascular structures in the wall of the ePCL tube was not hampered by the presence of an additional hydrogel on top. Whole mount immunofluorescence staining of a slice of the construct revealed the interwoven 3D structure of the EC network extending from the lumen through the ePCL tube wall into the additional hydrogel demonstrating the interaction between cells located in different hydrogels (Figure 1D, top and bottom middle). In addition, the presence of hollow structures was confirmed by confocal microscopy and 3D reconstruction (Figure 1D, bottom middle: 3D reconstruction, right: orthogonal views).

Compared to similar approaches [1, 2], which relied as well on self‐assembly of vascular networks and in addition on engineered pore architectures that restricted interconnections to predefined inlets, the porous ePCL tube used here provided a far larger surface for EC network integration, potentially enabling superior mass transport and extensive vascularization. Additionally, the overall size of the tubes (length: 60 mm, inner diameter: 3 mm) generated in our study is considerably larger compared to the tubes generated in the mentioned studies (length: 14 to 18 mm, inner diameter: 0.83 to 0.9 mm) [1, 2] rendering our approach unique in the field.

In summary, the combination of a highly porous ePCL tube, with the intrinsic ability of ECs to self‐assemble when co‐cultured with supporting cells in hydrogels, the addition of a second hydrogel, and the cultivation of the whole construct under perfusion resulted in the spontaneous organization of a vascularized structure throughout the entire construct. This system integrates the mechanical robustness and tunable architecture of synthetic scaffolds with the biological function of self‐assembled microvasculature. This achievement provides a promising platform for studying vascular network formation, anastomosis, and perfusion in vitro and is paving the way for a perfusable vascular matrix offering sites for surgical anastomosis and integration of other cell types for tissue reconstruction.

## Author Contributions

D.W. conducted and analyzed the experiments. M.G. conducted the experiments. A.E. contributed equipment and expertise in electrospinning. B.A. designed the study and wrote the manuscript. All authors approved the final version of the manuscript.

## Funding

This work was funded by zukunft.niedersachsen (Federal State of Lower Saxony), R2N.Micro‐Replace‐Systems.

## Ethics Statement

Patient material was processed following approval of the Ethics Committee at Hannover Medical School (file reference 3475‐2017) and after obtaining written informed consent from the patients. All tissues were used anonymously for this study. All experiments were performed in accordance with relevant guidelines and regulations.

## Conflicts of Interest

The authors declare no conflicts of interest.

## Supporting information




**Supporting File**: mco270985‐sup‐0001‐SuppMat.docx.

## Data Availability

The datasets generated during and/or analyzed during the current study are available from the corresponding author on reasonable request.
