# Supplementary material for: Electrospun Tubing as Shaping Material for Central Vessels in Perfusable, Vascularized Constructs
Source: MedComm (2020). 2026 Sep 8;7(9):e70985. doi: 10.1002/mco2.70985 (PMC13551341; doi:10.1002/mco2.70985)
Supplement: Supplementary file 1 [file MCO2-7-e70985-s001.docx]

Supporting Information

**Electrospun tubing as shaping material for central vessels in perfusable, vascularized constructs**

Daria Wehlage^a^, Mia Göcht^a^, Andrea Ehrmann^b^, Birgit Andrée^a^*

^a^Leibniz Research Laboratories for Biotechnology and Artificial Organs (LEBAO), Department of Cardiothoracic, Transplantation and Vascular Surgery, Hannover Medical School, Hannover, Germany

^b^ Faculty of Engineering and Mathematics, Bielefeld University of Applied Sciences and Arts, Bielefeld, Germany

**Correspondence to:**

Dr. Birgit Andrée (ORCID- iD: 0000-0001-6209-8240)

Leibniz Research Laboratories for Biotechnology and Artificial Organs (LEBAO),

Department of Cardiothoracic, Transplantation and Vascular Surgery, MHH

Carl Neuberg Str. 1

D-30625 Hannover, Germany

Phone:	+49 (511) 532 8913

FAX:	+49 (511) 532 8819

e-mail:	andree.birgit@mh-hannover.de

**Materials and Methods**

*Preparation of small intestinal submucosa (SIS)*

The preparation of decellularized small intestinal submucosa (SIS) was performed as previously described.^1-3^

*Human umbilical vein endothelial cells (HUVECs)*

HUVECs obtained from pooled donors were purchased from Lonza. Cells were cultured in Endothelial Growth Medium 2 (EGM-2, PromoCell). Passages 4-6 were used for all experiments.

*Isolation of adult human adipose tissue derived stromal cells (hASCs)*

Human adult adipose tissue-derived stromal cells (hASCs) were isolated from human tissues that were obtained from patients undergoing abdominoplasty. Isolation was performed according to a previously published protocol with minor modifications^4^. In brief, fat and connective tissue were minced, followed by addition of 10 mL Collagenase type II solution (760 U/mL, Worthington) per 20 mL of tissue and incubation for 1 hour at 37 ºC under shaking. Fatty supernatants were collected and washed with PBS. After centrifugation, the cell pellet was washed with PBS, resuspended and cells were cultivated in EGM-2. The medium was changed after 48 hours followed by a medium change every other day. Cells from passages 2-4 were used for the experiment.

*Preparation of lentiviral supernatants and cell transduction*

VSV.G-pseudotyped lentivirus particles were generated as described previously.^3^

### *Generation of electrospun tubes*

Electrospun PCL tubes were produced using the Fluidnatek Spinbox electrospinning system. A custom-made rotating mandrel (3 mm diameter, 150 mm length) served as the collector. The polymer solution was prepared by dissolving 1 g of PCL (M_n_: 80,000 g/mol) in 10 mL of a dichloromethane/chloroform mixture (3:7 v/v), which was stirred for 40 min at room temperature on a magnetic stirrer. The homogeneous polymer solution was loaded into a syringe connected via tubing to a 22 G blunt‑ended needle, which was attached to the high‑voltage power supply. Electrospinning was conducted at a voltage of 9.3 kV, a polymer flow rate of 1 mL/h, and a tip‑to‑collector distance of 20 cm. The mandrel rotation speed was maintained at 1000 rpm. During the electrospinning process, the temperature ranged between 21 and 23 °C, with a relative humidity of 30-35%. The electrospinning process was carried out for 2 h, after which the tube was gently removed from the mandrel by rinsing it with 70% ethanol to prevent fiber damage. The resulting PCL tube was air‑dried in an open petri dish at room temperature for at least 2 weeks to ensure complete solvent evaporation before use. Before being employed in cell culture experiments, PCL tubes were sterilized with 70% ethanol.

## *Characterization of the PCL matrix*

Segments from electrospun PCL tubes were cut open and sputtered with gold particles. The surfaces were visualized with a scanning electron microscope (SEM). Fiber diameters were determined in 6 images with ImageJ. In each of the 6 images, 50 fibers were randomly selected and manually measured. Mean fiber diameters and standard deviations (SDs) were calculated using GraphPad Prism. Wall thickness and porosity were quantified from cryosections. For wall thickness measurements, 12 tubes were analyzed (2 sections each with three measurements per section). Mean values and SDs were calculated using GraphPad Prism.

## *Sodium hydroxide treatment of the PCL tube*

Electrospun PCL tubes were equipped with Luer hose connectors, which were secured with Mersilene^TM^, to enable perfusion of the tube. To enhance the hydrophilicity of the electrospun PCL matrix, PCL tubes were treated with 1 M sodium hydroxide (NaOH) under continuous perfusion at a flow rate of 5 mL/min for 6 h. Subsequently, tubes were rinsed overnight with sterile water at a reduced flow rate of 2 mL/min to remove residual NaOH. Complete removal of NaOH was confirmed by determining the pH of the wash solution using pH indicator paper.

## *Hydrogel preparation*

## Hydrogels containing GFP-HUVECs and hASCs were based on rat tail collagen (rCOL) and prepared as previously published with minor modifications.^1,5^ In brief, 37.5 vol% Cultrex® 3‑D Culture matrix rat collagen I (3447-020-01, R&D Systems), 4.6 vol% double distilled H_2_O, and 24.4 vol% gel medium were mixed and neutralized with 8.1 vol% 0.4 M NaOH. The cell mixture resuspended in 25.4 vol% of EGM-2 medium was added and mixed with the hydrogel. A total of 13 × 10^6^ cells/mL with a ratio of 1:1.75 for HUVECs to hASCs were used. For polymerization, the respective constructs were incubated at 37 °C for 45 minutes.

## *Injection of hydrogel into the PCL tube*

A PCL tube was mounted onto a metal mold and a total volume of 570 µL cell-containing hydrogel was injected into the lumen of the PCL tube. Injection was stopped once the hydrogel reached the distal end of the tube and the outlet was subsequently sealed. To ensure complete infiltration of the hydrogel throughout the porous PCL matrix, the syringe was disconnected from the tube, and 300 µL of air was drawn up with the same syringe and injected into the lumen to push the hydrogel into the fiber network. A stainless-steel rod (2.4 mm diameter) was subsequently inserted into the lumen to maintain patency during polymerization. The PCL tube was maintained under static conditions for 24 h in EGM-2. The stainless-steel rod was carefully removed and the tube was bisected. One half was cultivated under static conditions for additional 21 days; the other half was cultivated under perfusion for 21 days with gradual increase of the flow rate from 0.25 mL/min to 1 mL/min within the first 6 days.

*Integration of electrospun PCL tubes in large vascularized constructs*

A dedicated bioreactor system was developed consisting of a base metal plate and metal holders to secure the decellularized SIS as a support matrix for the large vascularized construct. The bioreactor system assembly started with the base metal plate, which was also used as a mold for injecting a hydrogel into the PCL tube. The metal plate was laid out with decellularized SIS which was secured with metal holders on the plate. The electrospun PCL tube was placed onto the SIS within the groove of the base plate. The Luer hose connectors were inserted into dedicated housings and secured with two holders to maintain their position during injection and perfusion. PCL tubes were injected with a cell-containing hydrogel as described above. After 15 minutes a silicone mat with an oval-shaped cutout was positioned on top of the tube to define the casting area of the second hydrogel. This silicone mat was additionally stabilized by a rectangular metal holder, which was placed on top. A second cell‑containing hydrogel was prepared as described above and 550 µL were cast into the oval-shaped cutout of the silicon mat. After transfer into a custom-made glass chamber, the construct was connected to a glass capillary. After polymerization, the bioreactor chamber was filled with cultivation medium. Perfusion was initiated 24 h after casting at a flow rate of 0.25 mL/min, gradually increasing to 1 mL/min over the first 6 days. Constructs were cultivated for a total of 22 days.

*Immunofluorescence staining*

Whole-mounting immunofluorescence staining was performed according to standard protocols. In brief, tissues were fixed with 4% paraformaldehyde for 15 min at room temperature followed by three washing steps using TBS containing 10% Triton X-100 (TBSX). Samples were blocked 1 h at room temperature using 5% donkey serum in TBSX. Samples were incubation with primary anti‑CD31 antibody (1:500, ab9498, abcam) at room temperature for 48 h. The tissues were subsequently washed four times with TBSX for 5 min each before incubation with secondary antibody (Cy™3 AffiniPure® Donkey Anti-Mouse IgG (H+L), 1:300, 715-165-151, Jackson ImmunoResearch) for 48 h at room temperature. Subsequently, the samples were washed five times with TBSX. The samples were cleared employing ethyl cinnamate (protocol adapted from Dardano et al.^6^). Samples were imaged using confocal microscopy (Zeiss 980 Airyscan). 3D surfaces were automatically reconstructed using Imaris (version 9.3.1, Bitplane).

Alternatively, fixed samples were embedded in Tissue-Tek O.C.T. compound, frozen at -80 °C overnight, and sectioned. Sections were counterstained with 4′,6-diamidino-2-phenylindole (DAPI) (0.7 µg/mL in PBS) for 10 min at room temperature. After three additional washes with PBS‑Tween for 5 min each, the samples were mounted with fluorescence mounting medium and glass coverslips. Specimens were photographed using an Axio Observer 7 microscope (Zeiss).

*Documentation*

Living tissues were observed using the Stereo Microscope Discovery V8 (Zeiss).

*References*

1.	Manikowski D, Andree B, Samper E, et al. Human adipose tissue-derived stromal cells in combination with exogenous stimuli facilitate three-dimensional network formation of human endothelial cells derived from various sources. *Vascul Pharmacol*. 2018;106:28-36.

2.	Lux M, Andree B, Horvath T, et al. In vitro maturation of large-scale cardiac patches based on a perfusable starter matrix by cyclic mechanical stimulation. *Acta Biomater*. 2016;30:177-187.

3.	Vukadinovic-Nikolic Z, Andree B, Dorfman SE, et al. Generation of bioartificial heart tissue by combining a three-dimensional gel-based cardiac construct with decellularized small intestinal submucosa. *Tissue Eng Part A*. 2014;20(3-4):799-809.

4.	Strauss S, Dudziak S, Hagemann R, et al. Induction of osteogenic differentiation of adipose derived stem cells by microstructured nitinol actuator-mediated mechanical stress. *PLoS One*. 2012;7(12):e51264.

5.	Andrée B, Ichanti H, Kalies S, et al. Formation of three-dimensional tubular endothelial cell networks under defined serum-free cell culture conditions in human collagen hydrogels. *Sci Rep*. 2019;9(1):5437.

6.	Dardano M, Wilson L, Zweigerdt R, Drakhlis L. Production of human blood-generating heart-forming organoids and sample preparation for advanced imaging. *Nat Protoc*. 2026;21:2995-3025
